# The expression of IGFBP-5 in the reproductive axis and effect on the onset of puberty in female rats

**DOI:** 10.1186/s12958-022-00966-7

**Published:** 2022-07-12

**Authors:** Zhiqiu Yao, Maosen Lin, Tao Lin, Xinbao Gong, Pin Qin, Hailing Li, Tiezhu Kang, Jing Ye, Yanyun Zhu, Qiwen Hong, Ya Liu, Yunsheng Li, Juhua Wang, Fugui Fang

**Affiliations:** 1 Anhui Provincial Key Laboratory for Local Livestock and Poultry Genetic Resource Conservation and Bio-Breeding, 130 Changjiang West Road, Hefei, 230036 Anhui China; 2 Department of Animal Veterinary Science, College of Animal Science and Technology, Anhui Agricultural University, 130 Changjiang West Road, Hefei, 230036 Anhui China

**Keywords:** Insulin-like growth factor-binding protein-5 (IGFBP-5), Hypothalamus, Reproductive axis, Rat, Puberty

## Abstract

Insulin-like growth factor-binding protein-5 (IGFBP-5) has recently been shown to alter the reproductive capacity by regulating insulin-like growth factor (IGF) bioavailability or IGF-independent effects. The present study aimed to investigate the effect and mechanism of IGFBP-5 on the onset of puberty in female rats. Immunofluorescence and real-time quantitative PCR were used to determine the expression and location of IGFBP-5 mRNA and protein distribution in the infant's hypothalamus-pituitary-ovary (HPO) axis prepuberty, peripuberty, puberty and adult female rats. Prepubertal rats with IGFBP-5 intracerebroventricular (ICV) were injected to determine the puberty-related genes expression and the concentrations of reproductive hormones. Primary hypothalamic cells were treated with IGFBP-5 to determine the expression of puberty-related genes and the Akt and mTOR proteins. Results showed that *Igfbp-5* mRNA and protein were present on the HPO axis. The addition of IGFBP-5 to primary hypothalamic cells inhibited the expression of *Gnrh* and *Igf-1* mRNAs (*P* < 0.05) and increased the expression of AKT and mTOR protein (*P* < 0.01). IGFBP-5 ICV-injection delayed the onset of puberty, reduced *Gnrh*, *Igf-1*, and *Fshβ* mRNAs, and decreased the concentrations of E2, P4, FSH,serum LH levels and the ovaries weight (*P* < 0.05). More corpus luteum and fewer primary follicles were found after IGFBP-5 injection (*P* < 0.05).

**Supplementary Information:**

The online version contains supplementary material available at 10.1186/s12958-022-00966-7.

## Introduction

As a part of growth and development, puberty is the process of sexual maturation and the attainment of fertility. An elaborate neural network integrating many internal homeostatic and external signals governs the onset of puberty. These signal interactions ultimately result in increased synthesis and release of gonadotropin-releasing hormone (GnRH), and stimulation of GnRH leads to increased secretion of gonadotropins and complete gonadal maturity and function [1]. Studies over the past two decades have provided important information on the onset of puberty. The hormones, insulin, and insulin-like growth factor (IGF) network link to puberty [2–5]. The complex IGF network involves several growth factors (IGF-1, IGF-2), insulin-like growth factor-binding protein (IGFBP) and IGF receptors. Several studies have proved that the IGF network is considered pivotal in the upstream control of GnRH synthesis and secretion at puberty [2, 3, 6]. However, the effects of IGFBP on the onset of puberty have not been investigated thoroughly.

IGFBPs are highly homologous and have a high affinity with IGF. The IGFBPs alter the reproductive capacity by regulating IGF bioavailability or IGF-independent effects [7, 8]. As a part of IGFBPs, IGFBP-5 is widely distributed in the hypothalamus, pituitary, uterus, and ovary and is one of the major IGFBPs in the brain [9, 10]. In research using in situ hybridization to determine the temporal expression model of the IGF system, IGFBP-5 was shown to function directly in the growth and development of the rat anterior pituitary gland and have a specific temporal pattern of expression [11]. Females have higher levels of IGFBP-5 during the pubertal period in the anterior pituitary gland of female rats, which could be associated with the described effect of estrogen on the gene [12, 13]. Our previous study demonstrated that the levels of *Igfbp-5* mRNA in the hypothalamus are significantly different in pubescent and prepubescent goats [14]. These results indicated that IGFBP-5 might be a crucial regulator in the onset of female puberty.

In conclusion, we speculated that IGFBP-5 might influence reproductive hormonal changes and affect the onset of puberty. Therefore, in this study, we investigated (1) IGFBP-5 expression patterns in the hypothalamus-pituitary-ovary (HPO) axis; (2) the effects of IGFBP-5 on puberty onset, reproductive hormones and genes, and ovarian morphology in female rats; and (3) AKT and mTOR protein expression in hypothalamic cells.

## Materials and methods

### Animals

Adult Sprague Dawley (SD) rats were purchased from the Experimental Animal Center of Anhui Medical University. Adult SD rats were allocated into breeding pairs and allowed to deliver pups normally in the Anhui Agricultural University animal facility. The day of birth was considered postnatal day 1 (PND 1). Female pups were weaned at PND 20 and housed 4–5 per cage under controlled conditions of light (lights on at 06:00 and off at 18:00) and temperature (22 ± 1 °C), with ad libitum access to standard rat chow and water.

### Expression of IGFBP-5 in the HPO axis in rats at different periods

The hypothalamus, pituitary gland, and ovary were collected to measure Igfbp-5 gene levels (*n*=30) and immunolocalization of IGFBP-5 (*n*=30) in 60 female rats, which were euthanized with 2% pentobarbital sodium (50 mg/kg) at infant (PND 10, 10~16g, *n*=12), prepubertal (PND 21, 40~60g, *n*=12), peripubertal (PND 28, 50~80g, *n*=12), pubertal (about PND 34, 100~140g, *n*=12) and adult (PND 80, 190~230g, *n*=12) rats. Six rats of similar weights were randomly selected from each stage to assess *Igfbp-5* gene levels, and the remaining six animals at each stage were used to evaluate the IGFBP-5 immunolocalization. The hypothalamus was collected using dissection procedures described in a previous report [15]. The pituitary gland was removed from the skull, and the ovaries were removed via bilateral surgery. The timing of puberty onset in female rats was defined based on external vaginal opening (VO) [16].

### Fluorescence immunolocalization of IGFBP-5

The hypothalamus, pituitary, and the right ovary samples from 25 animals at five time periods (*n* = 6/time period) were fixed in 4% paraformaldehyde at 4 °C for 12 h embedded in paraffin to prepare 5 μm-thick sections. After being dehydrated with xylene and rehydrated with alcohol, the slices were subjected to antigen repair with sodium citrate buffer heated to 98 °C for 10 min [17]. Then the slides were blocked with 5% bovine serum albumin (Sigma, USA) in 1 × PBS for 1 h at 37 °C and incubated with rabbit anti-IGFBP5 antibodies (Omni Labs, USA) overnight at 4 °C. After washing three times with 1 × PBS, the slides were incubated with donkey anti-rabbit immunoglobulin (Ig) G-fluorochrome NL557 (1:200 dilution; NL010, R&D Systems, USA) at 37 °C for 1 h. Finally, the negative control group was treated with 1 μL secondary antibody alone. Sections were further incubated with DAPI (4′,6-diamidino-2-phenylindole) to stain nuclei. The sections were then imaged using a fluorescence microscope (OLYMPUS IX71, Germany). The mean fluorescence intensities of sections were analyzed by Image-Pro Plus 6.0 (Media Cybernetics, USA), and the mean fluorescence intensities were the result of cumulative optical density divided by total area according to Wang et al. [18] to quantify the positively stained area.

Briefly, five sections per animal were immunolabeled, and immunofluorescence pictures per region were acquired from Arcuate Nucleus (ARC), supraoptic nucleus and supraoptic decussation (SOR&SOX), anterior pituitary and ovary. Image-Pro software was used to analyze and quantify these photos in the following steps: (1) images are converted to grayscale 8 bit, thresholded, and the area of a target drawn for individual brain section was delineated; (2) the ratio of fluorescence intensity to the total area of the analyzed region is calculated to quantify the image of the designated brain area; (3) The immunoreactivity was represented by the Integrated Optical Density value (IOD)/Area.

### Study of onset of puberty, serum hormone concentrations, levels of puberty-related gene expression, and ovarian morphology following IGFBP-5 treatment

#### Experiment 1

Eighteen rats (PND25, 50~70g) were randomly divided into three groups: a control group (*n*=6), a 1 μg/kg group (*n*=6), and a 2 μg/kg group (*n*=6). The rats were administered an intracerebroventricular (ICV) injection of saline (control), 1 μg/kg IGFBP-5 (Creative BioMart, USA), or 2 μg/kg IGFBP-5 by skilled experimenters according to procedures from a previous study [19]. Two microliters injection volumes were used for all experiments. Briefly, 1% sodium pentobarbital solution (60 mg/kg, Sigma, USA) was used to anesthetize rats and deeply positioned in a stereotaxic apparatus. Under aseptic conditions, insert the microsyringe with 2 μl IGFBP-5 protein or saline at a 90° angle into a 2.4 mm posterior to the bregma and 0.5 mm lateral to the midline, 8.6 mm inferior to the skull [20]. The injection rate was 0.2 μL/min, and the syringe was kept in place for an additional 5 min to allow the injected solution to diffuse into the ARC prior to withdrawal [21]. After treatment, all rats were inspected daily (08:00-10:00; 15:00-16:00; 21:00-22:00), and the age at the vaginal opening (VO) was recorded. Once the VO was observed, the rats were euthanized to collect samples of the hypothalamus, pituitary gland, ovaries and blood according to the above methods in 2.2. The serum samples were collected (200 ×*g*, 20 min) and stored at −20°C until the enzyme-linked immunosorbent assay was performed. Hypothalami, pituitaries and the left ovaries of all experimental rats were collected and immediately frozen in liquid nitrogen and stored at -80 °C prior to extraction of RT-qPCR; the right ovaries were fixed with 4% paraformaldehyde and then embedded in paraffin. RT-qPCR was used to analyze mRNA levels of reproduction-related genes, including *Gnrh*, *Kiss-1*, *Igf-1r* and *Igf-1* in the hypothalamus, *Igfbp-5*, *Fshb*, *Igf-1*, *Igf-1r* and *Lhb* in the pituitary gland, and *Igf-1,Igf-1r* and *Igfbp-5* in the ovary. All rats were euthanized when the vaginal was opened, so this experiment was abbreviated as “all VO”(AVO). The objective of this experiment aimed to determine whether IGFBP-5 affects puberty onset and to analyze the physiological and morphological changes of rats during puberty.

#### Experiment 2

In this part, twelve 25d rats (50 ~ 70 g) were divided into an experimental group (*n* = 6) and control group (*n* = 6), which were respectively ICV injected with a 2μL IGFBP-5 solution (2 μg/kg) and saline. The ICV experiment method was the same as that described in 2.3.1. After ICV injection, rats of the control group were inspected daily and euthanized at VO.

At the same time, the rats in the experimental group were euthanized without opening the vagina. ***Experiment 1*** has shown that puberty onset was significantly delayed in prepuberty rats treated with IGFBP-5. Therefore, this experiment was abbreviated as “matched age” (MA). The sample collection and processing methods were the same as the AVO experiment, including blood samples, HPO tissues, and ovaries. This experiment is to study the differences in hormone levels, ovarian morphology and gene expression in rats of the same age after IGFBP-5 ICV injection.

#### Experiment 3

To study the estrous cycle and reproductive performance in prepubertal female rats following IGFBP-5 treatment, Twenty female rats (PND 25, 50~70g) were divided into two groups. The experimental group (*n*=10) was ICV injected in the hypothalamus with a 2μL IGFBP-5 solution (2μg/kg), and the control group (*n*=10) was injected with an equal volume of saline. The ICV method was the same as that described in 2.3.1*.* After VO, the estrous cycle of the rats was observed by vaginal smears taken from 9:00 to 11:00 every day for 18 consecutive days. On the 55th day after ICV injection, all female rats and additional adult male rats (PND 85, *n*=20, not part of the experimental cohort) were then mated according to a 1:1 ratio, and the breeding time was 21 days. Adult male rats can produce offspring in previous matings, indicating that they have no reproductive disorders. Determine the mating situation of rats by observed vaginal suppository, and female rats are observed every day (8:00-10:00; 15:00-16:00; 21:00-22:00) after pregnancy and record the litter size and offspring weight.

### Serum hormone assays

The progesterone (P_4_, KESHUN, China), estradiol (E_2_, KESHUN, China), follicle-stimulating hormone (FSH, KESHUN, China), luteinizing hormone (LH, KESHUN, China), and IGF-1 (KESHUN, China) in serum collected in experiment 2 were measured using rat commercial ELISA kits according to the manufacturer’s instructions (Wuhan Xinqidi Biological Technology, Wuhan, China) [22]. All assays were performed in triplicate. The sensitivity of the assay was 1.0 pg/ml for E_2_, 0.1 ng/ml for P_4_ and IGF-1, 1.0 IU/L for FSH, and 0.1 mIU/ml for LH. The intra-assay and inter-assay coefficients of variation of all kits were less than 10% and 15%. The standard curve coefficients of all hormones in the serum tested above were 0.9935 (E_2_), 0.9972 (P_4_), 0.9993 (FSH), 0.9932 (LH), and 0.9933 (IGF-1). No samples were below the limit of detection.

### Vaginal smears and ovarian morphology observation

The diameter and circumference of the right ovary (*n* = 6 ovary/group) obtained from rats in 2.3.1 and 2.3.2 were measured with a vernier calliper. Then, we fixed the ovarian tissues in 4% paraformaldehyde at 4 °C for over 12 h. Ovarian tissue samples were then embedded with paraffin and serially sectioned at 5 μm thickness. The sections were then deparaffinized in xylene, hydrated using a series of ethanol concentrations, and stained with hematoxylin and eosin (HE). Five sections of the ovary of each rat were selected to analyze the number of follicles and corpus luteum, with a total number of 30 sections/group.

In 2.3.3, vaginal lavage was performed daily (9:00–11:00) to determine the estrus cycle for 18 consecutive days after VO. Collected smears were mounted on glass slides and examined microscopically for cell type [23].

Studies on the expression of puberty-related genes and AKT-mTOR protein in primary hypothalamic cells after IGFBP-5 treatment.

We isolated primary hypothalamic cells from PND 1 female rats [24]. Briefly, rats were decapitated, and then the brains were rapidly removed and immersed in cold (4 °C) Dulbecco’s modified Eagle’s medium (DMEM) medium (Hyclone, USA). Hypothalamic tissue bounded by optic chiasma, lateral hypothalamic nuclei, and posterior margin of mammillary bodies was removed from 8 rats. The hypothalamic tissues were cut into fragments and incubated in 0.125% trypsin (Gibco, 25,200,056, USA) and DNase I (Biomgia, USA) for 20 min at 37 °C. Then, DMEM medium containing 10% fetal bovine serum (Hyclone, USA) was added, and the tissues were dissociated by mild mechanical trituration. About 1 × 10^5^ cells in 1 mL were plated onto poly-D-lysine-coated 6-well plates for further culture. After 12 h, the DMEM medium was replaced with Neurobasal™ medium (Gibco, 21,103,049, USA) supplemented with 2% B-27 serum-free supplement (Gibco, 17,504,044, USA) a humidified incubator containing 95% air and 5% CO_2_ at 37 °C. Primary cells were cultured to approximately 70% confluency (2 × 10^5^ cells per well) and then treated with IGFBP-5 at a dose of 25 or 50 ng/ml or the same volume of saline for 30 h. We conducted pre-experiments based on the literature on mice and human serum IGFBP-5 concentrations[25–27]. The results found that two dosages, 25 ng/ml and 50 ng/ml, significantly affected gene expression levels (data not shown). After 30 h, part of hypothalamic cells was collected for RNA isolation. RT-qPCR was used to analyze mRNA levels of reproduction-related genes, including *Gnrh, Kiss-1, Igf-1r* and *Igf-1* in the hypothalamus cells. Another part of the hypothalamic cells was lysed with RIPA buffer (Servicebio, G2002, China) supplemented with 1 × PMSF (Servicebio, G2008, China). Lysates were incubated on ice for 30 min and centrifuged at 12,000 × g for 10 min at 4 °C. The total protein concentration in the supernatant was determined by the BCA kit (Servicebio, G2026, China), denatured in a boiling water bath for 15 min and stored at -80 °C for western blot.

### Western blotting for AKT and mTOR protein

The above protein solution stored at -80 °C in 2.4 were resolved on a 10% SDS–polyacrylamide gel (SDS-PAGE). The separated proteins were electrophoretically transblotted onto Poly (vinylidene fluoride) membranes. After transfer, membranes were blocked with 5% nonfat dried milk/0.1% Tween 20 (Solarbio, T8220, China) in PBS (pH 7.4) for 1 h and subsequently incubated at 4 °C overnight with rabbit anti-Akt (1:1000; Proteintech, 10,176–2-AP, USA) or rabbit anti-mTOR (1:1000; Cell Signaling Technology, 2983, USA), and rabbit anti-actin (1:1000; Servicebio, GB12001, China). After the incubation, membranes were washed in PBS/0.1% Tween 20 and then incubated with horseradish peroxidase-labelled goat antirabbit IgG (1:3000; Servicebio, GB23303, USA) for 30 min at room temperature. After washing, the specific signals were detected by chemiluminescence (ECL, Servicebio, G2014, China). The strip's grey value was measured using alphaEaseFC (Alpha Innotech, USA) scanning camera.

### Total RNA isolation and RT-qPCR

According to the manufacturer's agreement, OMEGA E.Z.N.A.™ Total RNA Kit II (OMEGA, R6934, China) was used to extract total RNA from tissues from 2.2, 2.3.1, 2.3.2 and cells from 2.4. RNA purity was checked by spectrophotometer (NanoDrop 2000c, Thermo Scientific), and RNA integrity was checked by examining agarose gel electrophoresis. For RT-qPCR analysis, 500 ng total RNA was reverse-transcribed with EasyScript One-Step gDNA Removal and cDNA Synthesis SuperMix (Trans, Beijing, China) [21]. Primers of target genes were designed online using Primer 5 software and validated by BLAST [14]. The qRT-PCR was performed in triplicate with SYBR green Supermix (Vazyme, China) in a StepOnePlus Real-Time PCR System (ABI StepOnePlus 4,376,600, USA). The expression of the target gene was normalized to *GAPDH* and calculated using the comparative quantification method (2^−ΔΔCt^). All primer sequences are listed in Table 1. RT-qPCR analyses were carried out in triplicate from each rat in each group.

**Table 1:** Real-time PCR primers and sizes of the amplification products of the target genes in rat

Gene	Forward primer, 5′-3′	Reverse primer, 5′-3′	Product size,(bp)
*Igfbp-5*	CAGTCGTGTGGCGTCTAC	AGCGGCTTCTCCTCATCC	77
*Igf-1*	GCTGGTGGACGCTCTTCAGT	TTCAGCGGAGCACAGTACAT	168
*Gapdh*	TCACCACCATGGAGAAGGC	GCTAAGCAGTTGGTGGTGCA	134
*Fshβ*	TACTGCTACACCAGGGATCTG	TCTTACAGTGCAGTCGGTGC	201
*Lhβ*	GCTGCTGAGCCCAAGTGT	GCTGGTGGTGAAGGTGATG	124
*Igf-1r*	ACGCTGACCTCTGTTACCT	ATTGTTGATGGTGGTCTTCTC	161
*Kiss-1*	TGCTGCTTCTCCTCTGTG	CCAGGCATTAACGAGTTCC	116
*Akt*	CTCTTCTTCCACCTGTCTCG	ACAGCCCGAAGTCCGTTA	184
*Mtor*	GAGATACGCCGTCATTCC	GCATCAGAGTCAGGTGGTC	176
*Gnrh*	GCCGCTGTTGTTCTGTTGAC	CTGGGGTTCTGCCATTTGA	153

### Statistical analyses

For RNA samples generation, two hypothalamic tissues, pituitary or ovary, were pooled before RNA isolation, and the generated samples were processed independently. RT-qPCR analyses were conducted in triplicate. All data are expressed as mean ± SEM. Statistical Analyses All the statistical analyses were performed in SPSS version 19.0 (Chicago, USA). The results of the three groups were statistically analyzed using one-way ANOVA, whereas differences between the two groups were evaluated using Student’s t-test. A value of *P*<0.05 was considered significant. The differences between the two groups were compared by independent samples t-test. GraphPad 5.0 software (GraphPad, San Diego, CA, USA) was used to draw figures.

## Results

### Expression of the *Igfbp-5* mRNA and protein in hypothalamus, pituitary, and ovary from infant to adult rats

**Fig. 1:**
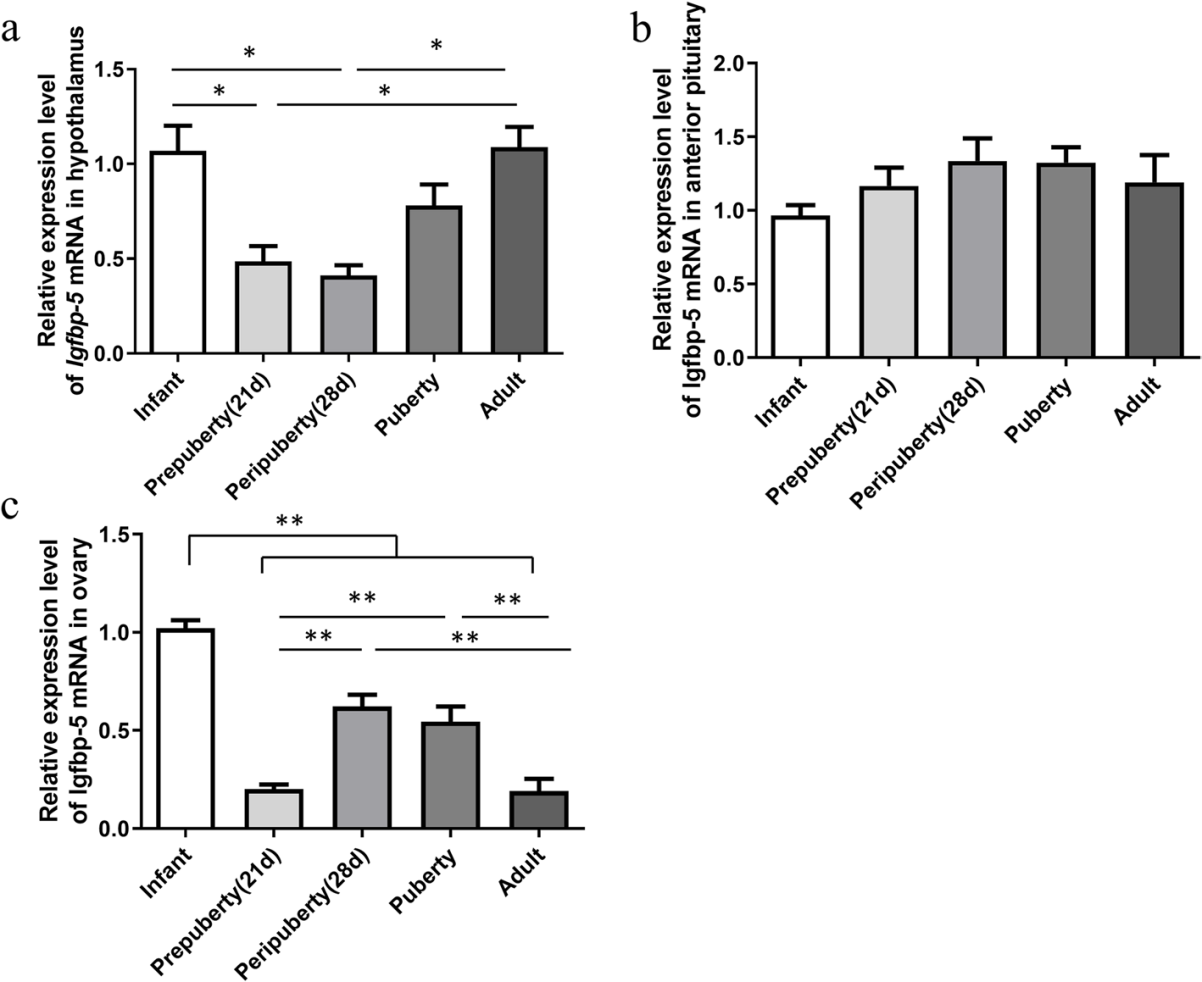
Level of *Igfbp-5*mRNA in the hypothalamus, anterior pituitary, and ovary of female rats from infancy to adult (*n* = 6/stage). **a** *Igfbp-5* mRNA level in the hypothalamus. **b** *Igfbp-5* mRNA level in the pituitary. **c** *Igfbp-5* mRNA levels in the ovary. ** indicates a very significant difference (*P* < 0.01); * indicates a significant difference (*P* < 0.05)

RT-qPCR revealed that the *Igfbp-5* mRNA level in the hypothalamus at prepuberty and peripuberty was significantly lower (*P* < 0.05) than in the infant. The *Igfbp-5* mRNA levels in the infant and adult rats are higher than in the other groups (Fig. 1a). No significant differences in pituitary *Igfbp-5* expression were observed from infant to adulthood (Fig. 1b). In the ovary, the expression level of *Igfbp-5* reached the highest level in infants, and after which, they decreased significantly at prepuberty (*P* < 0.01). The level of *Igfbp-5* mRNA in the ovary increases significantly during peripuberty and puberty (*P* < 0.01) and then decreases in adulthood (*P* < 0.01) (Fig. 1c).

**Fig. 2:**
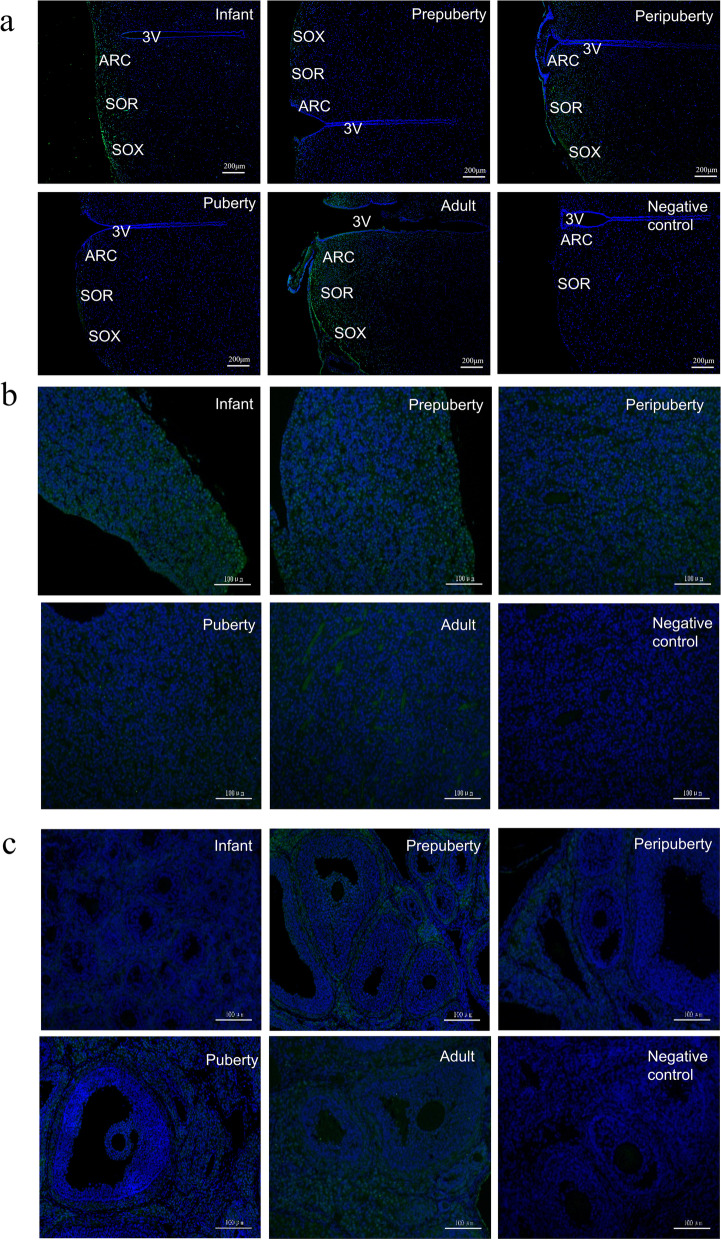
The distribution of the IGFBP-5 in the hypothalamus, anterior pituitary and ovary at the different developmental stages (*n* = 6/stage). **a** Localization of IGFBP-5 immunopositive cells in hypothalamus of female rats. cscale bar: 200 µm. **b** Localization of IGFBP-5 immunopositive cells in anterior pituitary of female rats. scale bar: 100 µm. **c** Localization of IGFBP-5 immunopositive cells in ovary of female rats. scale bar: 100 µm

**Fig. 3:**
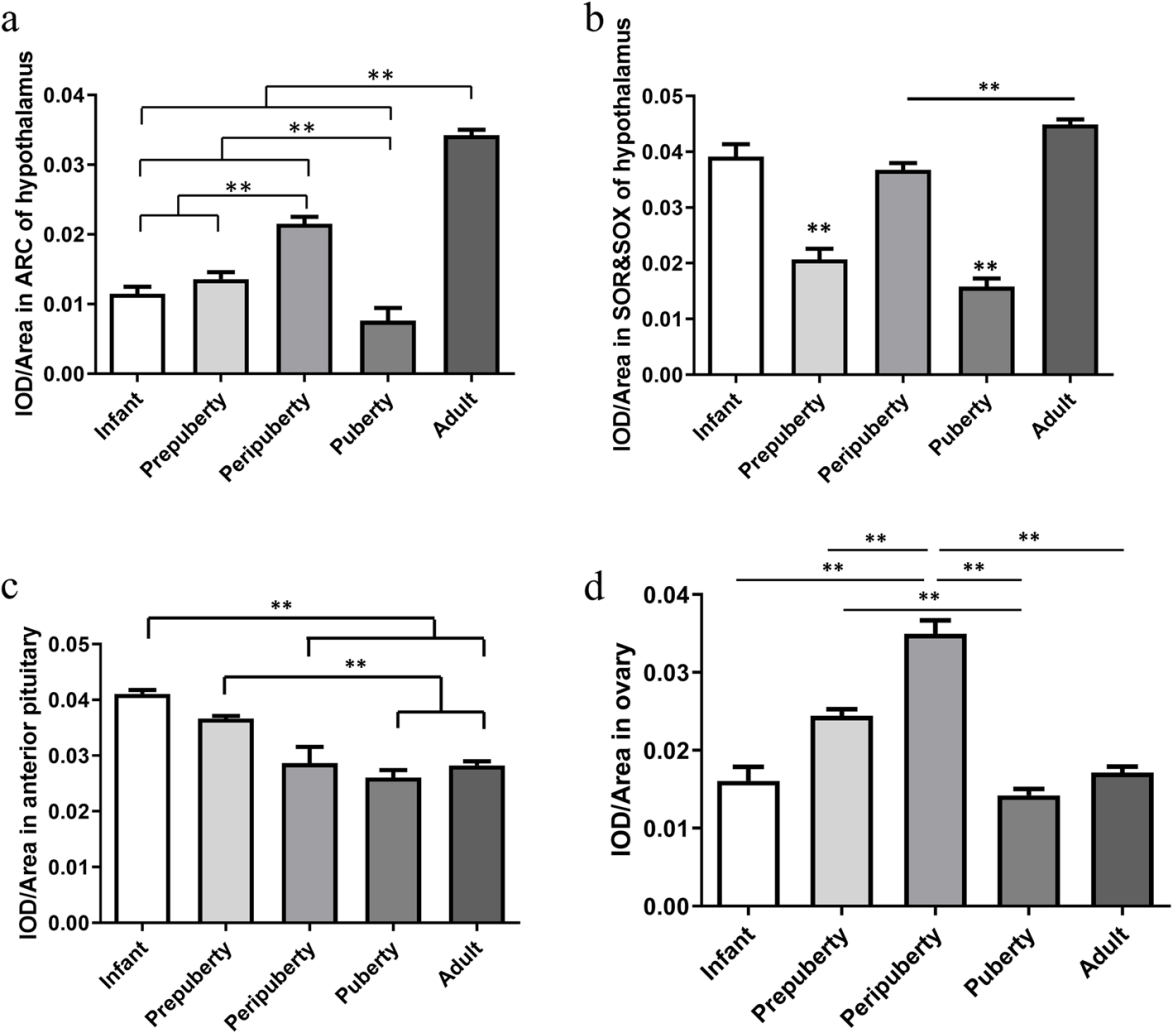
The mean fluorescence intensity of IGFBP-5 in the hypothalamus, pituitary and ovary of female rats at different developmental stages (*n* = 6/stage). **a **The IGFBP-5 mean fluorescence intensity in ARC of hypothalamus at the defferent developmental stages. **b **The IGFBP-5 mean fluorescence intensity in SOR&SOX of hypothalamus at the defferent developmental stages. **c** The IGFBP-5 mean fluorescence intensity in anterior pituitary of female rats at the defferent developmental stages. **d **The IGFBP-5 mean fluorescence intensity in ovary female rats at the defferent developmental stages. ** indicates a very significant difference (*P* < 0.01); * indicates a significant difference (*P* < 0.05)

IGFBP-5 immunoreactivity (IR) was detected in the ARC and SOR&SOX (Fig. 2a). Widespread distribution of IGFBP-5 protein in various rat anterior pituitary, revealed by Immunofluorescence analysis (Fig. 2b). IGFBP-5 is distributed in the ovarian stroma, granulosa cells, and theca cells in infant, prepubertal, peripubertal, pubertal and adult rats (Fig. 2c). The fluorescence intensity of IGFBP-5 in the SOR&SOX and ARC of pubertal rats was significantly lower than that in the peripubertal rats (*P* < 0.05) (Fig. 3a). Compared with the IGFBP-5 fluorescence intensity in the ARC and SOR&SOX nuclei in puberty, the fluorescence intensity of IGFBP-5 increases in adulthood. The IGFBP-5 fluorescence intensity in SOR&SOX was high at the infant, drastically decreased at the prepuberty stage (*P* < 0.01), and increased again at the peripuberty stage (*P* < 0.01) (Fig. 3b). The IGFBP-5 fluorescence intensity in the anterior pituitary gradually decreases with the development of rats (Fig. 3c). The fluorescence intensity of IGFBP-5 in the ovary reached the peak at peripuberty and then significantly declined at puberty (*P* < 0.05). IGFBP-5 in the ovary of adult rats also remained at a low level (Fig. 3d).

### Puberty-related gene expression and the time of vaginal opening in female rats after injecting IGFBP-5 in vivo

**Fig. 4:**
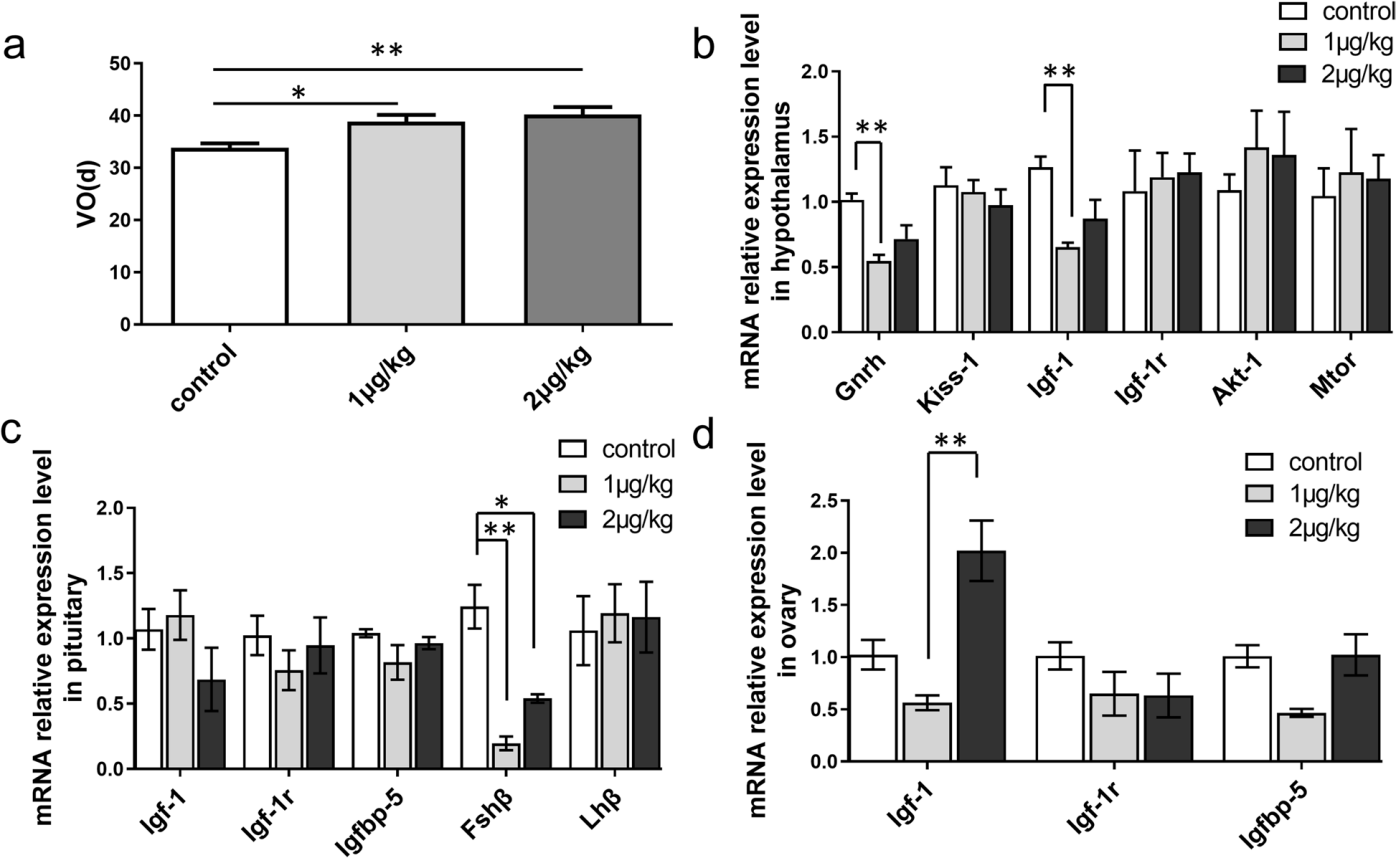
The effect of IGFBP-5 on puberty-relative gene expression and the time of vaginal opening in AVO experiments of female rats (*n *= 6/group). **a** The time of vaginal opening in female rats after IGFBP-5 ICV injection. **b** The expression level of puberty-related gene mRNA at VO in the rat hypothalamus (AVO). **c** The expression level of Igf-1, Igf-1r, Igfbp-5, Fshβ and Lhβ mRNA at VO in the rat pituitary (AVO). **d** The expression level of Igf-1,Igf-1r and Igfbp-5 mRNA at VO in the rat ovary (AVO). ** indicates a very significant difference (*P* < 0.01); * indicates a significant difference (*P* < 0.05)

As shown in Fig. 4a, puberty onset was significantly delayed in prepuberty rats treated with 1 μg/kg and 2 μg/kg IGFBP-5 (1 μg/kg, *P* < 0.05; 2 μg/kg*, P* < 0.01). In the AVO experiment (Fig. 4b), ICV with 1 μg/kg IGFBP-5 protein reduced the hypothalamus's *Gnrh* and *Igf-1* mRNA transcript levels. The expression levels of *Tgf-ß* were slightly, but not significantly, lower in animals treated with 1 μg/kg or 2 μg/kg IGFBP-5. Compared with the control group, *Fshβ* mRNA levels were significantly lower in the pituitary gland of the 1 μg/kg group (*P* < 0.01) and 2 μg/kg group (*P* < 0.05) (Fig. 4c). No significant differences were observed among the three groups in the expression of IGF-related genes in the pituitary gland and ovary (Fig. 4d).

**Fig. 5:**
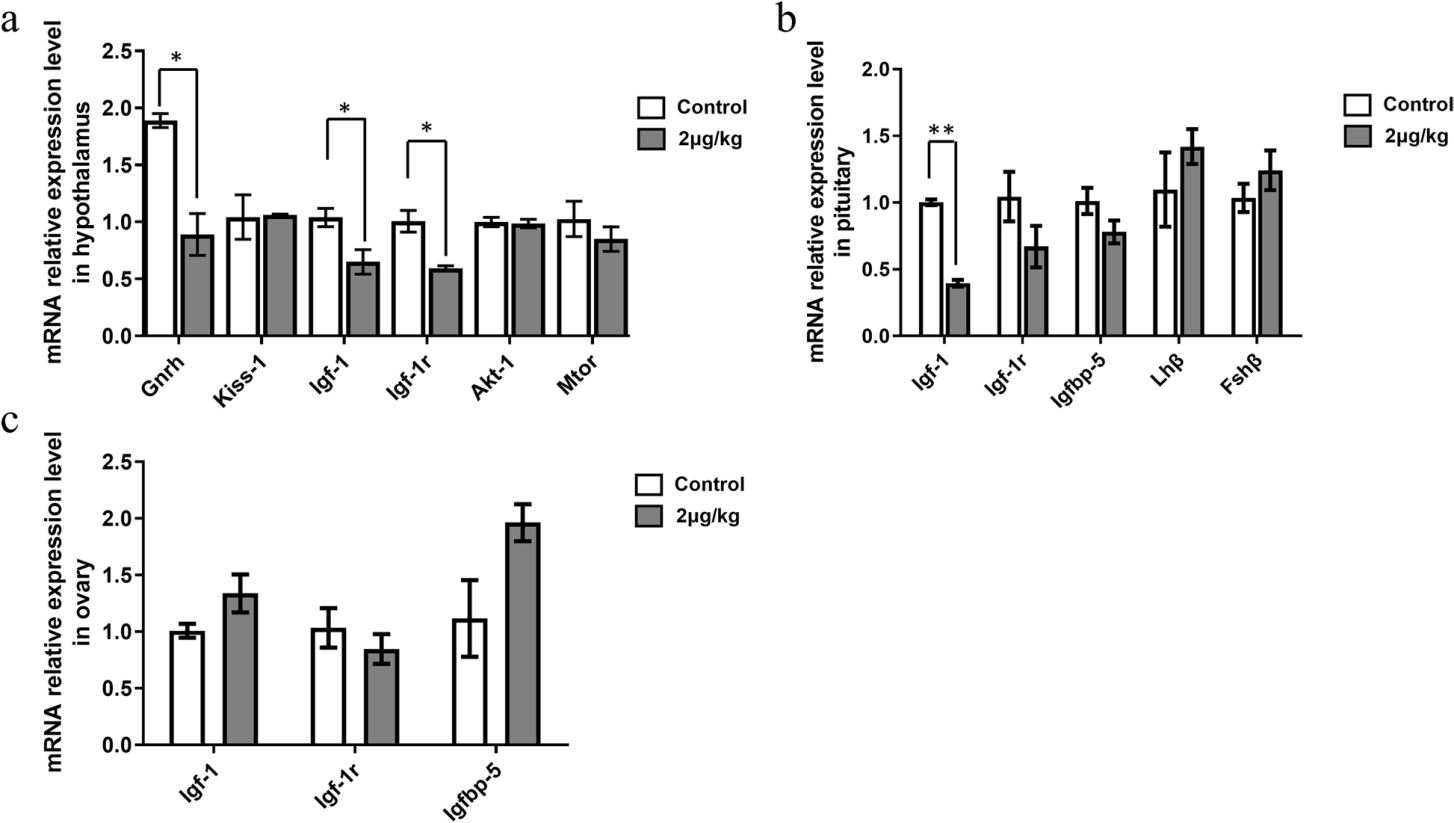
The effect of IGFBP-5 on puberty-relative gene expression and the time of vaginal opening in MA experiments of female rats (*n* = 6/group). **a** The expression level of puberty-related gene mRNA at matched age in the rat hypothalamus (MA). **b** The expression level of Igf-1,Igf-1r,Igfbp-5,Fshβ and Lhβ mRNA at matched age in the rat pituitary(MA). **c** The expression level of Igf-1,Igf-1r and Igfbp-5 mRNA at matched age in the rat ovary (MA). ** indicates a very significant difference (*P* < 0.01); * indicates a significant difference (*P* < 0.05)

In the MA experiment, The 2 μg/kg group showed a significantly lower mRNA expression level of *Igf-1r* and *Gnrh* in the hypothalamus than the control group (*P* < 0.05) (Fig. 5a). In addition, the *Igf-1* mRNA expression level in the pituitary gland was significantly lower in the 2 μg/kg group than in the control group (*P* < 0.01) (Fig. 5b). However, there was no significant difference in *Igfbp-5* mRNA levels between the two groups (Fig. 5c).

### Serum reproductive hormones and ovarian morphology in female rats after injecting IGFBP-5 in vivo

**Fig. 6:**
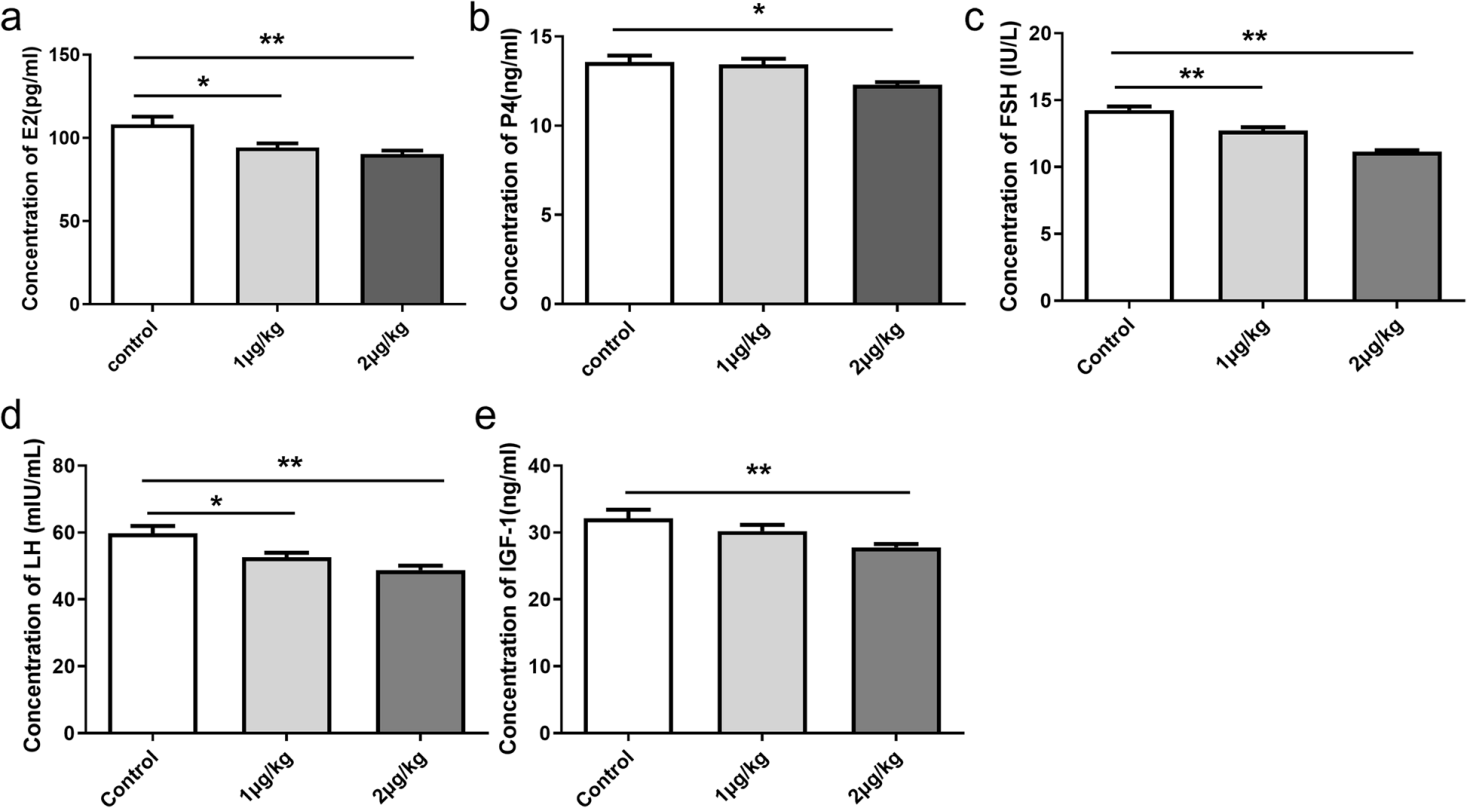
Effects of IGFBP-5 on serum hormone in AVO groups of female rats (*n *= 6/group) **a-e** The concentration of E2, P4, FSH, LH and IGF-1 in serum at VO after IGFBP-5 ICV injection (AVO). ** indicates a very significant difference (*P* < 0.01); * indicates a significant difference (*P* < 0.05)

**Fig. 7:**
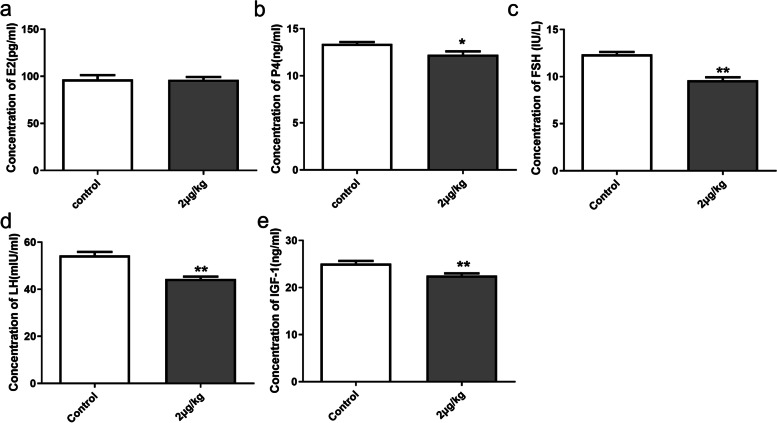
Effects of IGFBP-5 on serum hormone in MA groups of female rats (*n* = 6/group). **a-e** The concentration of E2, P4, FSH, LH and IGF-1 in serum at VO after IGFBP-5 ICV injection (MA). ** indicates a very significant difference (*P* < 0.01); * indicates a significant difference (*P* < 0.05)

As shown in Fig. 6, rats in the 2 μg/kg group showed significantly lower levels of serum IGF-1, E_2_, FSH, LH (both *P*<0.01) and significantly lower levels of P_4_ ( *P*<0.05) in comparison to the control group (Fig. 6a-e). In the MA experiment, no difference was observed in the concentration of E_2_ in serum (Fig. 7a). However, the concentrations of P_4_, FSH, LH and IGF-1 in the 2 μg/kg group were significantly lower than the control group (Fig. 7b-e).

**Fig. 8:**
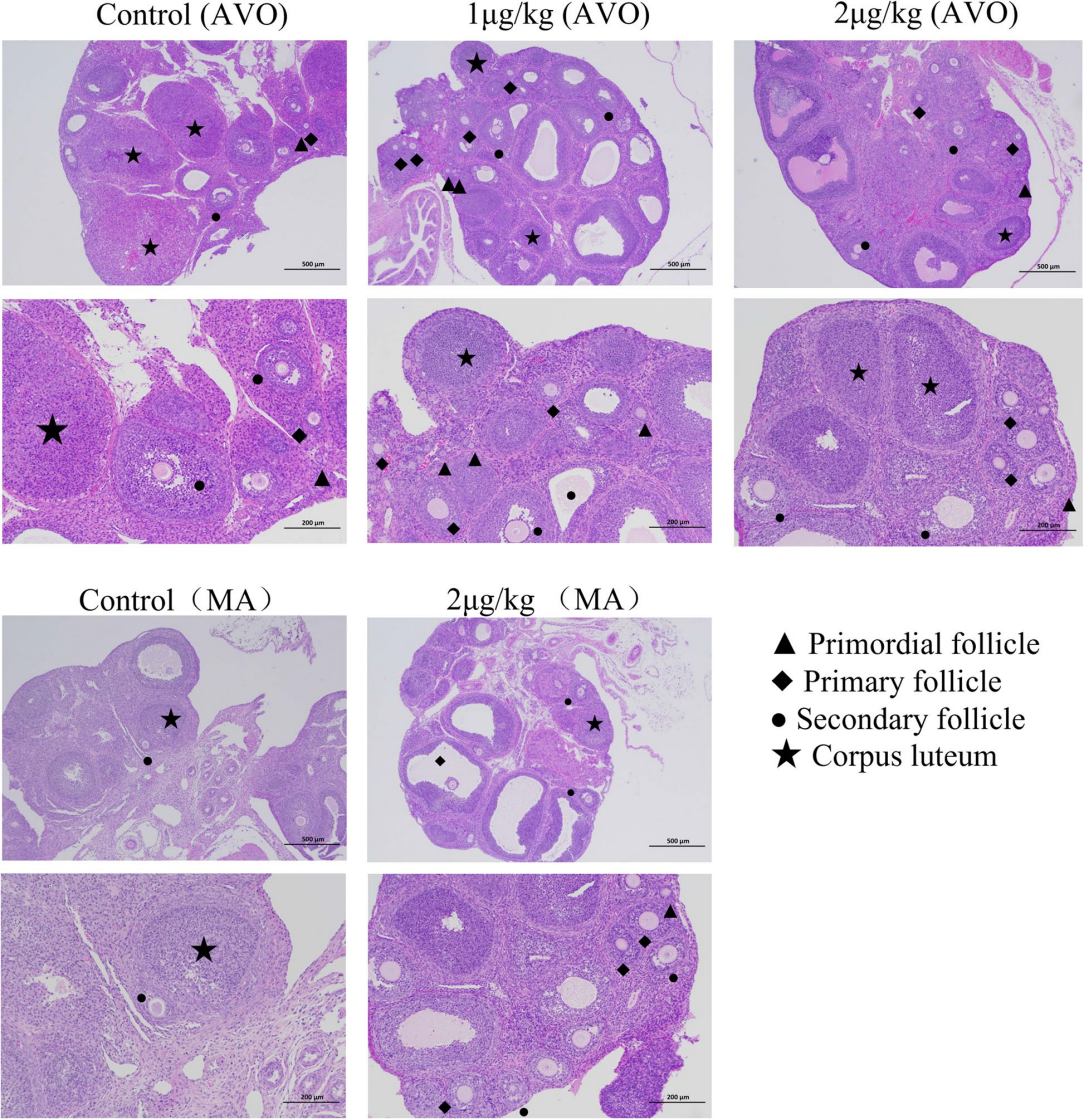
Effects of IGFBP-5 on ovarian morphology after ICV injection in female rats (*n* = 6/group). Histological assessment of follicles using H&E staining; ▲Primordial follicle; ◆ Primary follicle; ● Secondary follicles; ★Corpus luteum

In the AVO experiment, the ovarian weight was decreased by 1 μg/kg ICV at VO (*P* < 0.05); However, the size remained the same (Table 2). The results of HE staining showed that more primary follicles (*P* < 0.01) and fewer corpus luteum (*P* < 0.05) were found in the 1μg/kg group rats. Nevertheless, there was no significant difference in the number of secondary follicles among the three groups. The number of primary follicles in the 2μg/kg group was significantly more (*P* < 0.05), but the corpus luteum was less than the control group. Thus, the ovarian corpus luteum was reduced, and the number of small luminal follicles increased, which may cause a decrease in ovarian weight (Fig. 8).

**Table 2:** Effect of IGFBP-5 on morphology of ovary in female rat (n = 6/group) at VO

Group	Transverse diameter(cm)	Longitudinal diameter(cm)	Transverse perimeter(cm)	Longitudinal perimeter(cm)	Weight(g)	Primary Follicle (number)	Secondary Follicle (number)	Corpus Luteum (number)
Control	0.51 ± 0.06	0.37 ± 0.02	1.41 ± 0.10	1.02 ± 0.09	0.05 ± 0.01	2.33 ± 0.28	8.78 ± 1.37	4.22 ± 0.72
1 μg/kg	0.49 ± 0.05	0.36 ± 0.02	1.36 ± 0.07	1.02 ± 0.04	0.03 ± 0.01^*^	5.85 ± 0.91^**^	8.71 ± 1.89	2.28 ± 0.36^*^
2 μg/kg	0.50 ± 0.05	0.35 ± 0.02	1.35 ± 0.03	0.97 ± 0.07	0.04 ± 0.01	4.75 ± 0.75^*^	8.00 ± 0.86	2.77 ± 0.28

All data are shown as mean ± SEM. ** indicates a very significant difference (*P* < 0.01); * indicates a significant difference (*P* < 0.05)

In MA experiment, no significant difference was found in ovarian weight and size between the control group and the 2 μg/kg group in MA after IGFBP-5 injection. However, compared with the control group, more primary and secondary follicles were observed in the 2 μg/kg group (*P* < 0.05), and there was almost no corpus luteum in the 2 μg/kg group (*P* < 0.01) (Table 3).

**Table 3:** Ovarian weight and size of rats (n = 6/group) at match age after IGFBP-5 ICV injection

Group	Transverse diameter(cm)	Longitudinal diameter(cm)	Transverse perimeter(cm)	Longitudinal perimeter(cm)	Weight(g)	Primary Follicle (number)	Secondary Follicle (number)	Corpus Luteum (number)
Control	0.55 ± 0.22	0.35 ± 0.58	1.61 ± 0.28	1.02 ± 0.07	0.04 ± 0.01	3.62 ± 0.65	9.75 ± 0.86	4.28 ± 0.64
2 μg/kg	0.48 ± 0.12	0.32 ± 0.44	1.55 ± 0.01	0.90 ± 0.08	0.04 ± 0.01	4.40 ± 0.67^*^	11.00 ± 1.18^*^	0.50 ± 0.34^**^

All data are shown as mean ± SEM. ** indicates a very significant difference (*P* < 0.01); * indicates a significant difference (*P* < 0.05)

### The estrus cyclicity and weight of offspring in female rats after injecting IGFBP-5 in vivo

In experiment 3, after 2 μg/kg of IGFBP-5 protein was ICV injected, a vaginal smear test was performed to determine the estrus cycle after VO. No significant differences in the average estrus cycle were identified between the experimental group (4.348±0.223 d) and the control group (3.909±0.262 d) (Supplemental file 1). Similarly, there was no significant difference in the weight and number of offspring between the two groups (Table 4).

**Table 4:** The number and weight of offspring

Groups	Sex (*n* = 10)	number	Weight (g)
Control	Male	4.506 ± 2.014	6.596 ± 0.151
	Female	4.000 ± 0.949	6.591 ± 0.195
2 μg/kg IGFBP-5	Male	3.962 ± 1.772	6.402 ± 0.193
	Female	5.8 ± 1.356	6.552 ± 0.202

All data are shown as mean ± SEM

### Puberty-related gene expression and AKT-mTOR protein level in primary hypothalamic cells after IGFBP-5 treatment in vitro

**Fig. 9:**
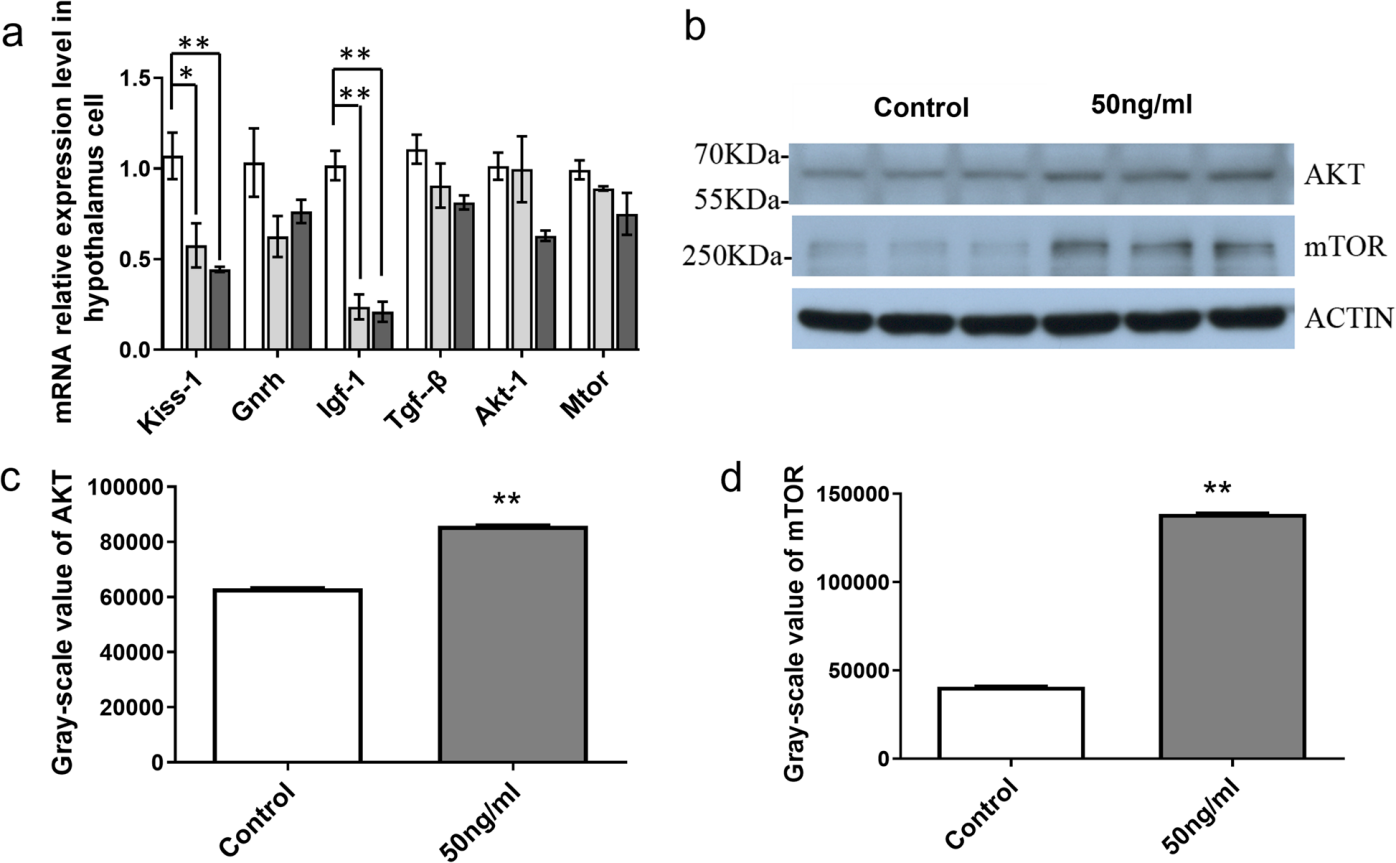
The effect of IGFBP-5 on gene expression and the AKT-mTOR protein in hypothalamus cells. **a** Levels of *Kiss-1, Gnrh, Igf-1, Tgf-β, Akt-1* and *Mtor* mRNA in hypothalamus cells after IGFBP-5 treatment. **b** Levels of AKT and mTOR protein in hypothalamus cells after IGFBP-5 treatment. **c** Relative gray scale of AKT expression determined following WB analysis. **d** Relative gray scale of mTOR expression determined following WB analysis.** indicates a very significant difference (*P* < 0.01); * indicates a significant difference (*P* < 0.05)

The expression levels of *kiss-1*, *Gnrh*, and *Igf-1* mRNA were decreased after adding different concentrations of IGFBP-5 (25 ng/ml, 50 ng/ml) protein to primary hypothalamic cells for 30 h (*P* < 0.05) (Fig. 9a). In addition, the expression levels of *Tgf-ß* and *Akt-1* mRNA decreased, although the difference was not significant.

To determine the effects of IGFBP-5 on the downstream pathway, we selected 50 ng/ml IGFBP-5 for WB; however, 50 ng/ml IGFBP-5 can more significantly reduce the expression of *Kiss-1* and *Igf-1* mRNA. Therefore, we assessed the effect of 50 ng/ml IGFBP-5 on the protein expression of AKT and mTOR by Western blot. The results demonstrate that IGFBP-5 caused an increase in AKT and mTOR protein (*P* < 0.01) (Fig. 9b, c, d).

## Discussion

### Expression of IGFBP-5 in the HPO axis of female rats at different development stages

Our study found that IGFBP-5 is present in tissues in the HPO axis. In the hypothalamus, IGFBP-5 was distributed in the hypothalamic nuclei, especially in the ARC, implicated in GnRH secretion [28, 29]. ARC is a key area for puberty initiation, and there are many neurons with positive Kiss-1 staining in the ARC and anteroventral periventricular nucleus involved in GnRH secretion [30]. In the present study, we found that *Igfbp-5* mRNA and protein expression exhibited a decreasing trend from prepubescence to puberty. In the brain, IGFBP-5 is one of the most highly expressed IGFBPs [31]. In addition, it has been reported that IGFBP-5 is involved in neuronal survival and plays a neuroendocrine function in the rat hypothalamus.

Furthermore, IGFBP-5expression is related to the content of IGF-I in specific brain regions [32]. Studies have also shown that the actions of IGF-1 are similar to the *kiss-1*/GnRH system at puberty and induce a precocious increase of *kiss-1* gene expression in the hypothalamus of prepubertal females rats [33, 34]. Given that IGFBP-5 plays a role in regulating IGF-1, This suggests that IGFBP-5 may modulate the secretion of hypothalamic hormones and regulate the onset of puberty.

The pars distalis (distal part) of the pituitary gland, where pituitary hormone production occurs, constitutes the majority of the anterior pituitary [35]. GnRH pulses stimulate the synthesis and secretion of LH and FSH from the anterior pituitary. Our results demonstrated that IGFBP-5 was primarily distributed in the anterior pituitary. It is the first study that deeply investigates the distribution of IGFBP-5 protein in the anterior pituitary at different development stages. IGFBP-5 is one of the most highly expressed binding proteins in the anterior pituitary in gene expression. At 10, 20, 30, or 60 days after birth, there was no significant change in the expression level of *Igfbp-5* mRNA in the pituitary of female rats, which is consistent with our results [36].

In our study, IGFBP-5 protein was also expressed in the ovarian stroma, granulosa cells, and theca cells, which are implicated in E_2_ and P_4_ secretion, indicating that IGFBP-5 may take part in regulating the secretion of steroid hormones. The IGFBP-5 fluorescence intensity in the ovary is the strongest in prepubertal rats and the faintest at puberty. Several studies have shown that cleavage of IGFBPs is enhanced by the increase of pregnancy-associated plasma protein-A (PAPP-A) proteolytic activity, which is modulated by IGFs [37, 38]. The increase of PAPP-A levels during puberty leads to an increase in the hydrolysis of IGFBP-5, which is related to the selection of dominant follicles [39]. Therefore, the IGFBP-5 degradation and PAPP-A increase could be linked [40, 41]. Although the *Igfbp-5* mRNA level was the highest in infants, there was no significant difference in expression levels in peripuberty and puberty. Thus, puberty is evaluated over a specified period rather than at a single point in time. *Igfbp-5* expression levels of the ovary increased markedly at peripuberty and maintained a constant level at puberty. This indicates that these changes occurred early in puberty, where gene expression was initiated but was not evident in the phenotype.

### The effect of IGFBP-5 on puberty in female rats

#### Puberty-related gene expression and the time of vaginal opening

In female rats, the time to the vaginal opening is commonly employed as a marker to determine the beginning of puberty [42]. A vast number of genes influence the regulation of vaginal opening time. In the present study, IGFBP-5 administration delayed the vaginal opening time and caused a marked suppression in *Gnrh* and *Igf-1* gene expression in the hypothalamus. IGF-1 is a key upstream signalling regulator of GnRH and onset of puberty and stimulates the expression of Kp protein in the same brain region [43, 44]. Interestingly, this study showed that IGFBP-5 lower the expression level of Igf-1 mRNA in the hypothalamus and lower the serum IGF-1 levels in female animals. Serum IGF-I increases before puberty. In previous studies concerning the *Igf-1* knockdown to block IGF signalling, it was also observed that the level of IGF-1 in serum was reduced [45, 46]. No change in expression of *Igf-1r* mRNA was observed in the MA experiment, whereas mRNA levels for *Igf-1r* mRNA were increased in the AVO experiment. The IGFBP-5 classical role is to regulate the availability and actions of the IGF-1. We believe that in the early stage after IGFBP-5 injection, IGFBP-5 binds the IGF-1 and prevents the activation of the IGF-1 receptor, which normally mediates cell signals to GnRH neurones [47]. The difference between the MA experiment and the AVO experiment is the length of time after the injection of IGFBP-5 in rats. The rats grew for approximately five more days because the time of VO was delayed in AVO. We consider that the inhibition of the IGF receptor may gradually weaken, presumably because of substantial compensation by remaining IGFBP family members [48, 49]. The global transgene overexpression of IGFBP-5 inhibited prenatal growth, especially before puberty, but low-dose overexpression did not significantly affect the fertility of female mice [49]. It is reasonable that IGFBP-5 and IGF-1, neuroendocrine genes that seem to regulate GnRH neuronal function, could function as part of the hypothetical “switch” that activates the GnRH neuronal network to initiate puberty. Exposure of female animals to endocrine disruptors or ICV injection of IGF-1 stimulator will affect the concentrations of IGF-1 and GnRH in the serum of female rats [50, 51]. The injection of IGFBP-5 reduces the concentration of serum IGF-1, which may be one of the reasons for the delayed puberty of female rats.

In the study, after IGFBP-5 injection, the level of *Lhβ* mRNA was not modified, whereas *Fshβ* mRNA was decreased in the pituitary. Furthermore, whereas E_2_ and LH serum levels were markedly decreased by central administration of IGFBP-5, there was no change in *Lhβ* mRNA. The apparent incongruity between the LH level and the levels of *Lhβ* mRNA that we have observed may be explained by the fact that *Lhβ* gene expression and release occur within a narrow range of GnRH pulse patterns and that variations on pulse pattern may be inhibitory exclusively at translational or posttranslational sites in the synthetic pathway of LH [52, 53]. Furthermore, different expression patterns of LH on mRNA and protein levels have been reported in the estrous cycle of adult dogs [54]. These data highlight the conflicting reports regarding LH and *Lhβ* in the literature.

### Serum reproductive hormones, ovarian morphology and the estrus cyclicity

In our study, the concentrations of FSH, LH, and IGF-1 in rat serum were reduced after an ICV injection of IGFBP-5. Considering that IGFBP-5 administration downregulated hypothalamic Gnrh gene expression levels, and the suppressed expression of GnRH was associated with decreased circulating levels of LH and FSH [33]. The suppressed circulating levels of these two hormones following IGFBP-5 injection were not surprising [55]. Puberty establishment is characterized by a progressive increase in the secretion of gonadotropic hormones that leads to an elevation in blood estradiol levels. The VO day and occurrence of the first estrus are markers of this event. In the present work, both parameters were delayed in females after IGFBP-5 injection. Delayed puberty usually occurs due to reduced estradiol levels resulting from either hypothalamic or pituitary dysfunction or damage in the ovary [56]. Our results confirm decreasing sex hormone levels, with no alteration in ovaries weights in females from the IGFBP-5 group at VO. Sex steroids are widely established to affect female reproductive organs' development, function, and differentiation [57]. During puberty, E_2_ and P_4_ enhance the reproductive endocrine activity of the hypothalamus and pituitary while releasing gonadotropin through negative feedback [58]. In the ovaries, on the other hand, an increase in the number of primary and secondary follicles was observed in IGFBP-5-injected females, despite their normal organ weight. Fewer corpus luteum was found in the IGFBP-5 injection rats. However, the level of E_2_ did not change significantly in MA. In mammals, E_2_ secretion rises in the late follicular phase during puberty in females [59]. The function and structure of the ovary gradually improve during puberty, and the content of E_2_ and P_4_ in the blood increases during the first ovulation and maintains the subsequent estrous cycle [60]. The more the primary and secondary follicles in the ovary may increase E_2_ secretion. The increase in the number of primary follicles and the decrease in the corpus luteum in the experimental group indicated that that follicular development was partially arrested [61].

Metestrus, estrus, proestrus, and diestrus are the four stages of the rat estrous cycle [20]. Progression of the estrous cycle as influenced by the HPG axis is another sign of normal female reproductive development [62]. In the present study, there was no significant difference in the estrous cycle. Thus, changes in the HPO axis may only occur in the period between prepuberty and puberty in females from the IGFBP-5 group, but they were re-established during adulthood, maintaining normal serum levels of progesterone and estradiol. Reproductive performance of rats was not affected by IGFBP-5 injection, suggesting that IGFBP-5 did not affect reproductive performance or litter weight. The ovaries still had normal physiological functions after puberty.

### The effect of IGFBP-5 on primary hypothalamic cells

In this study, the addition of IGFBP-5 protein to primary neurons in the hypothalamus similarly downregulated *Gnrh*, *Kiss-1*and *Igf-1* expression levels, which were identical to the results of ICV injections of IGFBP-5. The *Kiss-1* gene of the hypothalamus binds and activates the G protein-coupled receptor 54 (GPR54) to regulate the synthesis and secretion of GnRH, and then regulates the onset of puberty through the HPO axis. Thus, IGFBP-5 may function on the HPO axis by regulating *Kiss-1* of GnRH neurons during puberty. IGF-1 has been shown to up-regulate the *Kiss-1* gene and Kp expression in the AVPV and preoptic area/rostral hypothalamic area (POA/RHA), and this process is mediated by the Akt-mTOR pathway [2, 3, 63]. The complexity of IGFBP-5 actions *in vitro*, which may inhibit or potentiate IGF actions and exert IGF-independent actions, makes inference of physiological roles of this protein from these studies difficult. This is a controversial area with some contradictory findings [64]. The function of IGFBP-5 includes retaining IGF and enhancing its effect [65, 66]. In addition to IGF, IGFBP-5 can interact with other biomolecules, such as the epithelial extracellular matrix. The effect of IGFBP5 on cell adhesion is independent of IGF, and there is a positive correlation between IGFBP-5 and AKT was noted [67, 68]. In the nervous system, growth hormone significantly increased *Igf-1* mRNA levels in the central nervous system of mice, accompanied by an increase in IGFBP-5 protein and activation of the PI3K-AKT pathway [69]. In the present study, we demonstrated that the addition of IGFBP-5 increased AKT and mTOR in hypothalamic cells. This suggests that IGFBP-5 may partially affect the progression of puberty through the AKT and mTOR pathways. In vivo, IGFBP-5 inhibits *Igf-1* mRNA expression and decreases IGF-1 in serum, but in vitro, due to the disappearance of serum IGF-1, we hypothesized that there is an IGF-independent action of IGFBP-5. At the same time, IGFBP-5 also suppressed the production of endogenous IGF-1 in primary hypothalamic cells [70].In addition, it has been suggested that IGFBP-5 expression might be regulated by epigenetic mechanisms [71]. Polycomb group complex (PcG)-mediated epigenetic silencing may be an important regulatory mechanism of IGFBP-5 suppression in the pubertal rat [72]. Enhancer of zeste homologue 2 (EZH2) in the pleiotropic repressor complex 2 (PRC2) has gained considerable attention for its regulatory role in the maintenance of pluripotency [71], neural stem cell differentiation [72] and epigenetics [73]. EZH2 is a classical epigenetic regulator gene, and a genome-wide transcriptome study showed that the target gene of EZH2 in endothelial cells is IGFBP-5 and leads to epigenetic silencing of IGFBP-5 [71]. In cancer cell biology, it has been shown that PcG activates the AKT-mTOR signaling pathway through nuclear transcription factors(Ecotropic viral integration site 1) [74]. In breast cancer cells, lysine(K)-specific demethylase 6B and PI3K/AKT were able to promote IGFBP5 expression by suppressing EZH2-mediated histone three lysine 27 tri-methylation at the IGFBP5 promoter [75].These studies suggest a possible link between epigenetic silencing of IGFBP-5 and regulation of signaling pathways. Considering the findings of AKT-mTOR in this study, determining the expression of EZH2 in the hypothalamus during different physiological periods in subsequent studies, will provide further evidence supporting the epigenetic regulation of IGFBP-5 during puberty.

In summary, IGFBP-5 was expressed in the hypothalamus, pituitary and ovaries of female rats at different developmental stages, and the level of *Igfbp-5* mRNA in the HPO axis varied with the development process. Furthermore, after injection of IGFBP-5, the initiation of puberty was delayed, transcript levels of *Igf-1*, *Kiss1* and *Gnrh* mRNA in the hypothalamus were altered, and serum reproductive hormone concentrations were reduced. These results suggest that IGFBP-5 plays an essential role in the initiation of puberty in female rats.

## Supplementary Information

**Additional file 1.**

**Additional file 2.**

## Data Availability

All data are included in this article and its additional files.
