# Supplementary material for: The expression of IGFBP-5 in the reproductive axis and effect on the onset of puberty in female rats
Source: Reprod Biol Endocrinol. 2022 Jul 12;20:100. doi: 10.1186/s12958-022-00966-7 (PMC9277959; doi:10.1186/s12958-022-00966-7)
Supplement: Supplementary file 1 — Additional file 1. [file 12958_2022_966_MOESM1_ESM.doc]

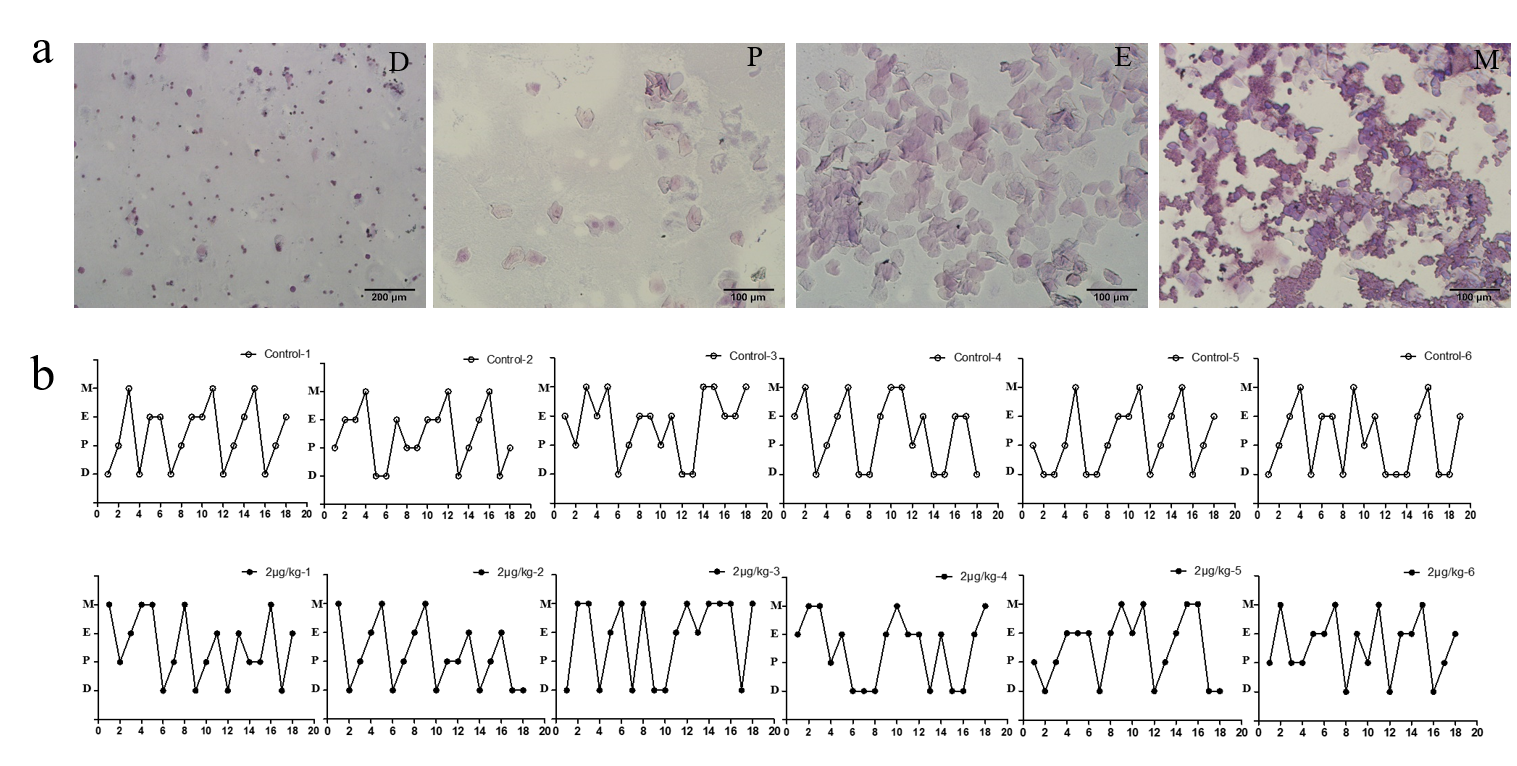


Effects of IGFBP-5 on estrus cyclicity after ICV injection. (a) Vaginal cell smears in diestrus, proestrus, estrus and metestrus. (b) Estrus cycle in rats after ICV injection of 2 μg/kg IGFBP-5 protein. M, Metestrus; E, estrus; P, proestrus; D, diestrus.
