# Supplementary figures and images for: The expression of IGFBP-5 in the reproductive axis and effect on the onset of puberty in female rats
Source: Reprod Biol Endocrinol. 2022 Jul 12;20:100. doi: 10.1186/s12958-022-00966-7 (PMC9277959; doi:10.1186/s12958-022-00966-7)

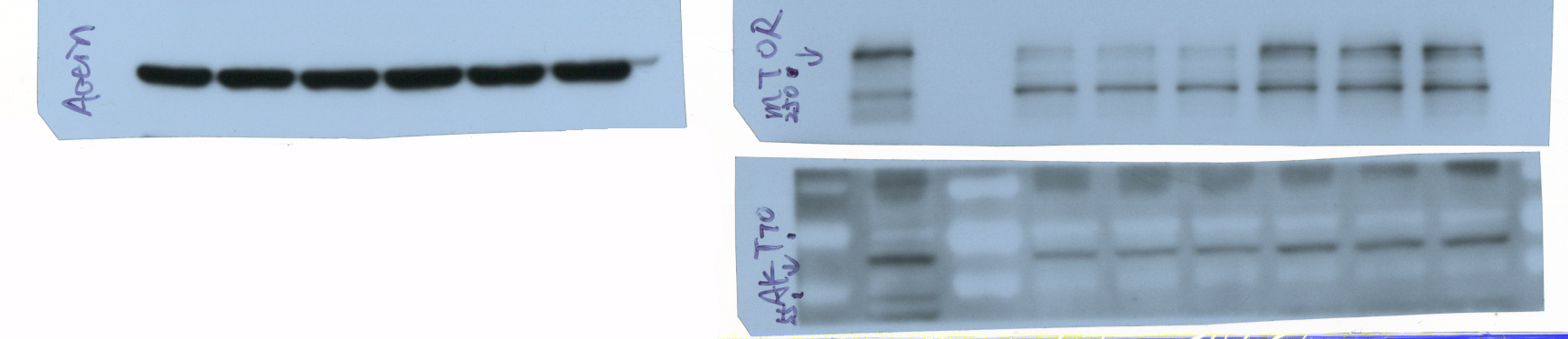


The original image of Western blotting.

Supplement: Supplementary file 2 — Additional file 2. [file 12958_2022_966_MOESM2_ESM.doc]
